# 2-(4-Chloro­anilino)-1-(4-chloro­phen­yl)ethanone

**DOI:** 10.1107/S1600536811048276

**Published:** 2011-11-19

**Authors:** Hoong-Kun Fun, Ching Kheng Quah, A. M. Vijesh, A. M. Isloor, T. Arulmoli

**Affiliations:** a X-ray Crystallography Unit, School of Physics, Universiti Sains Malaysia, 11800 USM, Penang, Malaysia; b SeQuent Scientific Ltd, No. 120 A & B, Industrial Area, Baikampady, New Mangalore, Karnataka 575 011, India, and, Medicinal Chemistry Division, Department of Chemistry, National Institute of Technology-Karnataka, Surathkal, Mangalore 575 025, India; c Medicinal Chemistry Division, Department of Chemistry, National Institute of Technology-Karnataka, Surathkal, Mangalore 575 025, India

## Abstract

In the title compound, C_14_H_11_Cl_2_NO, the benzene rings form a dihedral angle of 3.14 (6)°. Overall, the mol­ecule is close to being planar (r.m.s. deviation for all the non-H atoms = 0.054 Å). No significant directional inter­molecular inter­actions are observed in the crystal structure.

## Related literature

For general background to amine derivatives, see: Sridharan *et al.* (2006 ▶). For bond-length data, see: Allen *et al.* (1987 ▶). For related structures, see: Fun *et al.* (2010 ▶, 2011 ▶).
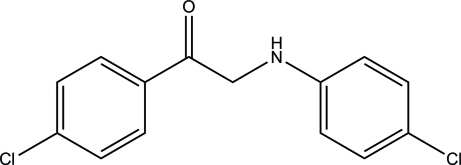


## Experimental

### 

#### Crystal data

C_14_H_11_Cl_2_NO
*M* *_r_* = 280.14
Triclinic, 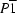

*a* = 5.7286 (3) Å
*b* = 7.4225 (5) Å
*c* = 15.4274 (9) Å
α = 85.337 (1)°
β = 89.772 (1)°
γ = 82.519 (1)°
*V* = 648.23 (7) Å^3^
*Z* = 2
Mo *K*α radiation
μ = 0.49 mm^−1^
*T* = 296 K
0.55 × 0.26 × 0.15 mm

#### Data collection

Bruker SMART APEXII DUO CCD diffractometer
Absorption correction: multi-scan (*SADABS*; Bruker, 2009 ▶) *T*_min_ = 0.776, *T*_max_ = 0.931
14634 measured reflections
4406 independent reflections
3279 reflections with *I* > 2σ(*I*)
*R*_int_ = 0.017

#### Refinement

*R*[*F* ^2^ > 2σ(*F* ^2^)] = 0.038
*wR*(*F* ^2^) = 0.121
*S* = 1.05
4406 reflections
167 parameters
H atoms treated by a mixture of independent and constrained refinement
Δρ_max_ = 0.29 e Å^−3^
Δρ_min_ = −0.31 e Å^−3^

### 

Data collection: *APEX2* (Bruker, 2009 ▶); cell refinement: *SAINT* (Bruker, 2009 ▶); data reduction: *SAINT*; program(s) used to solve structure: *SHELXTL* (Sheldrick, 2008 ▶); program(s) used to refine structure: *SHELXTL*; molecular graphics: *SHELXTL*; software used to prepare material for publication: *SHELXTL* and *PLATON* (Spek, 2009 ▶).

## Supplementary Material

Crystal structure: contains datablock(s) global, I. DOI: 10.1107/S1600536811048276/hb6501sup1.cif

Structure factors: contains datablock(s) I. DOI: 10.1107/S1600536811048276/hb6501Isup2.hkl

Supplementary material file. DOI: 10.1107/S1600536811048276/hb6501Isup3.cml

Additional supplementary materials:  crystallographic information; 3D view; checkCIF report

## Figures and Tables

**Fig. 1.**
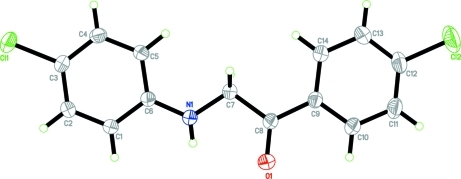
The molecular structure of the title compound showing 30% probability displacement ellipsoids for non-H atoms.

## supplementary crystallographic information

### Comment

1-(4-Chlorophenyl)-2-[(4-chlorophenyl)amino]ethanone is a derivative of an amine formed by the reaction between an amine and phenacyl bromide. It finds applications in the field of synthetic chemistry and is a key intermediate in indole synthesis (Sridharan *et al.*, 2006). Keeping this in view, the title compound was synthesized to study its crystal structure.

The molecular structure is shown in Fig. 1. The phenyl rings (C1-C6 and C9-C14) form a dihedral angle of 3.14 (6) °. Bond lengths (Allen *et al.*, 1987) and angles are within normal ranges and are comparable to related structures (Fun *et al.*, 2010, 2011). No significant hydrogen bond is observed in this compound.

### Experimental

The mixture of 4-chloroaniline (1.0 g, 0.0078 mol) potassium carbonate (0.95 g, 0.0069 mol) and 2-bromo-1-(4-chlorophenyl)ethanone (1.83 g, 0.0078 mol) in dimethyl formamide (10 ml) was stirred at room temperature for 2 h. On cooling, colorless needle-shaped crystals 2-(4-chlorophenyl)- 2-oxoethyl 2-methylbenzoate begins to separate. It was collected by filtration and recrystallized from hot ethanol as colourless needles. Yield: 1.85 g, 84.5 %, *m.p.*: 427-428 K.

### Refinement

H1N1 was located in a difference Fourier map and allowed to refine freely [N1–H1n1 = 0.780 (17) Å]. The remaining H atoms were positioned geometrically and refined using a riding model with C–H = 0.93 or 0.97 Å and *U*_iso_(H) = 1.2 *U*_eq_(C).

### Crystal data

**Table:**

C_14_H_11_Cl_2_NO	*Z* = 2
*M**_r_* = 280.14	*F*(000) = 288
Triclinic, *P*1	*D*_x_ = 1.435 Mg m^−^^3^
Hall symbol: -P 1	Mo *K*α radiation, λ = 0.71073 Å
*a* = 5.7286 (3) Å	Cell parameters from 5676 reflections
*b* = 7.4225 (5) Å	θ = 2.7–31.5°
*c* = 15.4274 (9) Å	µ = 0.49 mm^−^^1^
α = 85.337 (1)°	*T* = 296 K
β = 89.772 (1)°	Needle, colorless
γ = 82.519 (1)°	0.55 × 0.26 × 0.15 mm
*V* = 648.23 (7) Å^3^	

### Data collection

**Table:**

Bruker SMART APEXII DUO CCD diffractometer	4406 independent reflections
Radiation source: fine-focus sealed tube	3279 reflections with *I* > 2σ(*I*)
graphite	*R*_int_ = 0.017
φ and ω scans	θ_max_ = 31.9°, θ_min_ = 2.7°
Absorption correction: multi-scan (*SADABS*; Bruker, 2009)	*h* = −8→8
*T*_min_ = 0.776, *T*_max_ = 0.931	*k* = −10→10
14634 measured reflections	*l* = −22→22

### Refinement

**Table:**

Refinement on *F*^2^	Primary atom site location: structure-invariant direct methods
Least-squares matrix: full	Secondary atom site location: difference Fourier map
*R*[*F*^2^ > 2σ(*F*^2^)] = 0.038	Hydrogen site location: inferred from neighbouring sites
*wR*(*F*^2^) = 0.121	H atoms treated by a mixture of independent and constrained refinement
*S* = 1.05	*w* = 1/[σ^2^(*F*_o_^2^) + (0.0546*P*)^2^ + 0.1078*P*] where *P* = (*F*_o_^2^ + 2*F*_c_^2^)/3
4406 reflections	(Δ/σ)_max_ = 0.001
167 parameters	Δρ_max_ = 0.29 e Å^−^^3^
0 restraints	Δρ_min_ = −0.31 e Å^−^^3^

### Special details

**Table:**

Geometry. All esds (except the esd in the dihedral angle between two l.s. planes) are estimated using the full covariance matrix. The cell esds are taken into account individually in the estimation of esds in distances, angles and torsion angles; correlations between esds in cell parameters are only used when they are defined by crystal symmetry. An approximate (isotropic) treatment of cell esds is used for estimating esds involving l.s. planes.
Refinement. Refinement of F^2^ against ALL reflections. The weighted R-factor wR and goodness of fit S are based on F^2^, conventional R-factors R are based on F, with F set to zero for negative F^2^. The threshold expression of F^2^ > 2sigma(F^2^) is used only for calculating R-factors(gt) etc. and is not relevant to the choice of reflections for refinement. R-factors based on F^2^ are statistically about twice as large as those based on F, and R- factors based on ALL data will be even larger.

### Fractional atomic coordinates and isotropic or equivalent isotropic displacement parameters (Å^2^)

**Table:**

	*x*	*y*	*z*	*U*_iso_*/*U*_eq_	
Cl1	0.53272 (7)	0.38195 (5)	0.30213 (2)	0.06177 (13)	
Cl2	1.09875 (12)	0.23470 (8)	−0.52931 (3)	0.0986 (2)	
O1	1.46099 (17)	0.11247 (15)	−0.11538 (6)	0.0595 (3)	
N1	1.1416 (2)	0.20038 (17)	0.00358 (7)	0.0479 (3)	
C1	1.0802 (2)	0.20624 (16)	0.15684 (7)	0.0414 (2)	
H1A	1.2343	0.1513	0.1664	0.050*	
C2	0.9398 (2)	0.24941 (18)	0.22701 (8)	0.0450 (3)	
H2A	0.9990	0.2233	0.2833	0.054*	
C3	0.7107 (2)	0.33154 (16)	0.21332 (8)	0.0418 (2)	
C4	0.6222 (2)	0.37011 (17)	0.13004 (8)	0.0448 (3)	
H4A	0.4682	0.4259	0.1213	0.054*	
C5	0.7621 (2)	0.32599 (17)	0.05920 (8)	0.0428 (2)	
H5A	0.7008	0.3513	0.0032	0.051*	
C6	0.99495 (19)	0.24365 (14)	0.07159 (7)	0.0363 (2)	
C7	1.0671 (2)	0.23121 (16)	−0.08528 (7)	0.0392 (2)	
H7A	1.0121	0.3597	−0.0986	0.047*	
H7B	0.9371	0.1631	−0.0946	0.047*	
C8	1.2670 (2)	0.17276 (15)	−0.14502 (7)	0.0399 (2)	
C9	1.2203 (2)	0.19003 (15)	−0.24018 (7)	0.0392 (2)	
C10	1.3956 (2)	0.12290 (19)	−0.29565 (9)	0.0519 (3)	
H10A	1.5399	0.0686	−0.2726	0.062*	
C11	1.3597 (3)	0.1352 (2)	−0.38450 (9)	0.0623 (4)	
H11A	1.4780	0.0889	−0.4212	0.075*	
C12	1.1463 (3)	0.2168 (2)	−0.41788 (9)	0.0583 (4)	
C13	0.9689 (3)	0.2858 (2)	−0.36467 (9)	0.0592 (4)	
H13A	0.8257	0.3412	−0.3883	0.071*	
C14	1.0057 (2)	0.27170 (19)	−0.27579 (8)	0.0497 (3)	
H14A	0.8861	0.3172	−0.2394	0.060*	
H1N1	1.266 (3)	0.146 (2)	0.0124 (11)	0.058 (5)*	

### Atomic displacement parameters (Å^2^)

**Table:**

	*U*^11^	*U*^22^	*U*^33^	*U*^12^	*U*^13^	*U*^23^
Cl1	0.0600 (2)	0.0724 (2)	0.0522 (2)	−0.00031 (16)	0.01890 (15)	−0.01462 (16)
Cl2	0.1357 (5)	0.1203 (4)	0.03485 (19)	0.0016 (4)	−0.0021 (2)	−0.0062 (2)
O1	0.0450 (5)	0.0820 (7)	0.0464 (5)	0.0127 (5)	−0.0019 (4)	−0.0088 (5)
N1	0.0406 (5)	0.0649 (7)	0.0342 (5)	0.0077 (5)	0.0009 (4)	−0.0032 (4)
C1	0.0368 (5)	0.0484 (6)	0.0370 (5)	0.0015 (4)	−0.0030 (4)	−0.0028 (4)
C2	0.0460 (6)	0.0537 (7)	0.0343 (5)	−0.0022 (5)	−0.0024 (4)	−0.0050 (5)
C3	0.0422 (6)	0.0426 (6)	0.0409 (5)	−0.0038 (4)	0.0072 (4)	−0.0075 (4)
C4	0.0354 (5)	0.0496 (6)	0.0472 (6)	0.0009 (5)	0.0017 (4)	−0.0003 (5)
C5	0.0382 (5)	0.0511 (6)	0.0370 (5)	−0.0008 (5)	−0.0029 (4)	0.0007 (4)
C6	0.0370 (5)	0.0370 (5)	0.0346 (5)	−0.0034 (4)	0.0006 (4)	−0.0028 (4)
C7	0.0390 (5)	0.0440 (6)	0.0342 (5)	−0.0033 (4)	0.0020 (4)	−0.0044 (4)
C8	0.0402 (5)	0.0407 (5)	0.0382 (5)	−0.0017 (4)	0.0028 (4)	−0.0051 (4)
C9	0.0424 (5)	0.0389 (5)	0.0360 (5)	−0.0036 (4)	0.0041 (4)	−0.0042 (4)
C10	0.0497 (7)	0.0587 (7)	0.0442 (6)	0.0056 (6)	0.0078 (5)	−0.0063 (5)
C11	0.0715 (9)	0.0697 (9)	0.0429 (7)	0.0045 (7)	0.0163 (6)	−0.0099 (6)
C12	0.0788 (10)	0.0608 (8)	0.0348 (6)	−0.0071 (7)	0.0034 (6)	−0.0044 (5)
C13	0.0592 (8)	0.0745 (9)	0.0408 (6)	0.0020 (7)	−0.0048 (6)	−0.0024 (6)
C14	0.0463 (6)	0.0611 (8)	0.0393 (6)	0.0028 (5)	0.0028 (5)	−0.0056 (5)

### Geometric parameters (Å, °)

**Table:**

Cl1—C3	1.7406 (12)	C5—H5A	0.9300
Cl2—C12	1.7332 (14)	C7—C8	1.5091 (15)
O1—C8	1.2187 (14)	C7—H7A	0.9700
N1—C6	1.3738 (15)	C7—H7B	0.9700
N1—C7	1.4290 (15)	C8—C9	1.4855 (16)
N1—H1N1	0.780 (17)	C9—C10	1.3860 (16)
C1—C2	1.3798 (16)	C9—C14	1.3921 (17)
C1—C6	1.3985 (15)	C10—C11	1.3808 (19)
C1—H1A	0.9300	C10—H10A	0.9300
C2—C3	1.3823 (17)	C11—C12	1.374 (2)
C2—H2A	0.9300	C11—H11A	0.9300
C3—C4	1.3774 (17)	C12—C13	1.377 (2)
C4—C5	1.3881 (17)	C13—C14	1.3817 (18)
C4—H4A	0.9300	C13—H13A	0.9300
C5—C6	1.4000 (16)	C14—H14A	0.9300
			
C6—N1—C7	122.77 (10)	N1—C7—H7B	109.5
C6—N1—H1N1	120.3 (13)	C8—C7—H7B	109.5
C7—N1—H1N1	116.5 (13)	H7A—C7—H7B	108.1
C2—C1—C6	121.18 (10)	O1—C8—C9	121.31 (11)
C2—C1—H1A	119.4	O1—C8—C7	120.43 (10)
C6—C1—H1A	119.4	C9—C8—C7	118.26 (10)
C1—C2—C3	119.77 (11)	C10—C9—C14	118.66 (11)
C1—C2—H2A	120.1	C10—C9—C8	119.16 (11)
C3—C2—H2A	120.1	C14—C9—C8	122.18 (10)
C4—C3—C2	120.29 (11)	C11—C10—C9	121.18 (13)
C4—C3—Cl1	120.13 (9)	C11—C10—H10A	119.4
C2—C3—Cl1	119.56 (9)	C9—C10—H10A	119.4
C3—C4—C5	120.22 (11)	C12—C11—C10	118.92 (13)
C3—C4—H4A	119.9	C12—C11—H11A	120.5
C5—C4—H4A	119.9	C10—C11—H11A	120.5
C4—C5—C6	120.41 (10)	C11—C12—C13	121.39 (13)
C4—C5—H5A	119.8	C11—C12—Cl2	119.56 (12)
C6—C5—H5A	119.8	C13—C12—Cl2	119.05 (12)
N1—C6—C1	119.28 (10)	C12—C13—C14	119.30 (13)
N1—C6—C5	122.59 (10)	C12—C13—H13A	120.4
C1—C6—C5	118.13 (10)	C14—C13—H13A	120.4
N1—C7—C8	110.63 (10)	C13—C14—C9	120.55 (12)
N1—C7—H7A	109.5	C13—C14—H14A	119.7
C8—C7—H7A	109.5	C9—C14—H14A	119.7
			
C6—C1—C2—C3	−0.17 (19)	O1—C8—C9—C10	5.16 (19)
C1—C2—C3—C4	0.11 (19)	C7—C8—C9—C10	−174.56 (11)
C1—C2—C3—Cl1	178.85 (10)	O1—C8—C9—C14	−174.82 (13)
C2—C3—C4—C5	0.29 (19)	C7—C8—C9—C14	5.46 (17)
Cl1—C3—C4—C5	−178.44 (10)	C14—C9—C10—C11	−0.3 (2)
C3—C4—C5—C6	−0.64 (19)	C8—C9—C10—C11	179.70 (13)
C7—N1—C6—C1	178.89 (11)	C9—C10—C11—C12	0.5 (2)
C7—N1—C6—C5	−1.74 (19)	C10—C11—C12—C13	−0.1 (3)
C2—C1—C6—N1	179.23 (12)	C10—C11—C12—Cl2	179.61 (12)
C2—C1—C6—C5	−0.16 (18)	C11—C12—C13—C14	−0.3 (3)
C4—C5—C6—N1	−178.81 (12)	Cl2—C12—C13—C14	179.92 (12)
C4—C5—C6—C1	0.57 (18)	C12—C13—C14—C9	0.5 (2)
C6—N1—C7—C8	179.16 (11)	C10—C9—C14—C13	−0.2 (2)
N1—C7—C8—O1	−1.37 (17)	C8—C9—C14—C13	179.82 (13)
N1—C7—C8—C9	178.35 (10)		
